# From Task Distributions to Expected Paths Lengths Distributions: Value Function Initialization in Sparse Reward Environments for Lifelong Reinforcement Learning

**DOI:** 10.3390/e27040367

**Published:** 2025-03-30

**Authors:** Soumia Mehimeh, Xianglong Tang

**Affiliations:** School of Computer Science and Technology, Harbin Institute of Technology, 92 West Dazhi Street, Nangang District, Harbin 150001, China; tangxl@hit.edu.cn

**Keywords:** reinforcement learning, lifelong learning, statistical reinforcement learning, value function initialization

## Abstract

This paper studies value function transfer within reinforcement learning frameworks, focusing on tasks continuously assigned to an agent through a probabilistic distribution. Specifically, we focus on environments characterized by sparse rewards with a terminal goal. Initially, we propose and theoretically demonstrate that the distribution of the computed value function from such environments, whether in cases where the goals or the dynamics are changing across tasks, can be reformulated as the distribution of the number of steps to the goal generated by their optimal policies, which we name the *expected optimal path length*. To test our propositions, we hypothesize that the distribution of the expected optimal path lengths resulting from the task distribution is normal. This claim leads us to propose that if the distribution is normal, then the distribution of the value function follows a log-normal pattern. Leveraging this insight, we introduce “LogQInit” as a novel value function transfer method, based on the properties of log-normality. Finally, we run experiments on a scenario of goals and dynamics distributions, validate our proposition by providing an a dequate analysis of the results, and demonstrate that LogQInit outperforms existing methods of value function initialization, policy transfer, and reward shaping.

## 1. Introduction

Lifelong learning in reinforcement learning (RL) [1] is a transfer learning paradigm that involves continuously assigning various tasks to an agent, and it should learn while accumulating knowledge to improve its performance when faced with new tasks [2,3,4]. These tasks are drawn from a probabilistic distribution designed to reflect scenarios the agent is likely to encounter in real-world applications [5,6,7,8]. In practice, the distribution of tasks can design different types of changes in the environmental settings, mainly changes in dynamics and changes in the reward. For example, dynamics changes in applications can be seen as a mechanical or an electrical noise in robot sensors, which usually model a Gaussian distribution [7,8], or wind disturbances affecting a drone, where wind typically follows a Weibull distribution [9]. Tasks can also differ in terms of rewards and goals, such as in a delivery problem where the agent must deliver to customers in different locations that are spread across a city, which are typically distributed according to a normal distribution [5].

One effective strategy for transferring knowledge to new tasks is to initialize the agent’s function before it begins learning [3,10,11]. The objective of initialization is to provide knowledge in the early stages of learning in order to enhance both the initial, referred to as the jump-start [3,12], and the overall performance of the agent. The main challenge is therefore to estimate the value function of the new task as accurately as possible using data from previous tasks, in order to reduce the number of samples needed for training new tasks and enabling the agent to reach optimal solutions more quickly. Following this strategy, the agent is seen as if it were trying to predict the value function without interacting with the new environment. In machine learning, predictions are often based on the expected values of a dataset, which can be more accurately estimated when the parameters of the dataset distribution are known. Thus, it is essential to extract information from the distribution of RL tasks to accurately estimate the expected value function.

Previous research has explored using probabilistic information from task distributions, typically tackling empirical distributions through simple averaging [11] or through optimistic initialization of the value function based on hyper-parameters within the probably approximately correct Markov decision process framework [10,13]. On another hand, probabilistic information has been explored for experience data at the meta-reinforcement learning scale for policy transfer in deep reinforcement learning [14,15,16]. However, to our knowledge, there has been no research on how to specifically induce the connection between the distribution of the value functions from task distributions in this regard.

Therefore, our research focuses on initializing the value function in lifelong learning using the parameters of value function distribution. We specifically examine tasks within the framework of a terminal goal state and sparse reward. We claim that the state-action space remains similar across tasks while rewards or dynamics change. Our main idea is based on the insight that since the state-action space is the same for each task, the key difference between different task solutions lies in the policy (and hence the value function), which determines the set of actions needed to reach the goal. If the policies differ, then the sequence of states and actions leading to the goal (referred to as a “path”) will also differ in length. We use this insight to propose how to measure and deduce the value function distribution. We mainly provide the following contributions:

- We theoretically demonstrate that for tasks sharing the same state-action space, differences in goals or dynamics can be represented by multiple separate distributions of each state action, which is the distribution of the number of steps required to reach the goal, referred to as the *“Expected Optimal Path Length”*. We then show that the value function’s distribution of each state action is directly connected to its distribution of the expected optimal path lengths.
- We propose a method for initializing the value function in sparse reward settings by using a normal distribution of tasks. Our empirical experiments validate this approach by confirming our proposition about the distribution of the expected path lengths.

The rest of the paper is structured as follows: Section 2 reviews related works, while Section 3 summarizes the relevant preliminaries of RL. Section 4 introduces the definition of the expected optimal path length. In Section 5, we formulate the initialization problem and examine how the value function distribution is connected to the distribution of path lengths in both goal distributions and dynamics distributions. We then proceed to present a case study on the normal distribution of path lengths in Section 6, which serves as the foundation for our proposed initialization algorithm, LogQInit. Finally, Section 7 presents our experiments and results.

## 2. Related Works

**Knowledge Transfer:** Our research is situated within the broad domain of knowledge transfer in reinforcement learning, as outlined in the literature [2,3,4]. Knowledge transfer methods encompass a spectrum of approaches, ranging from policy transfer [17] to network parameters, experience data [18], and trajectory samples [19]. Our work focuses on value function initialization, aiming at the objective of enhancing the jump-start performance of the agent [12]. While the field of transfer reinforcement learning has seen limited exploration of value function initialization, existing studies have shown strong evidence that adequate initialization improves learning at the level of single tasks [20,21] and is, therefore, useful in transfer learning. We categorize two main axes of studies on value initialization as knowledge transfer: one explores value function transfer in environments with different state-action spaces and different dynamics by computing the bisimulation between target and source task reward and transition functions, and transferring the value between states with small distances [22,23,24]. However, these methods require full access to the environment model, which is not always known to the agent. Additionally, these approaches are known for being computationally intensive and only applicable to a limited number of tasks. In contrast, our method depends on task distribution information only and therefore eliminates the need for extensive computations, providing significant results that make it suitable for lifelong learning. Another axis of initialization methods explores the use of optimistic initialization theory; Ref. [10] proposed an optimistic initialization strategy based on the probably approximately correct framework [13] parameters to estimate the number of steps that should be learned from scratch before starting the transfer. Meanwhile, ref. [11] suggests that the optimistic initialization idea can be improved by trading between the maximum and the mean of the value function from previous tasks using confidence and uncertainty quantities within the agent parameters. However, these works only studied empirical and uniform distributions and did not track the exact relationships between the value function and the task distribution. To tackle this limitation, our work’s objective is to analyze the nature of the value function distribution according to the task distribution.

**Goal based Sparse Reward Tasks:** Since our study addresses challenges in a specific type of environment characterized by sparse rewards and a terminal goal, it shares similarities with a body of research known as goal-conditioned reinforcement learning [25,26]. In these scenarios, the agent’s objective is to reach a specified goal, with rewards provided exclusively upon reaching the goal. This makes the reward signals sparse and difficult to utilize effectively for learning.

Existing research typically tackles such environments through two main approaches. The first approach treats them as single-task problems with dynamic rewards, employing techniques like generating imaginary goals to augment the training data [27] or reshaping the reward function to provide more frequent feedback [28]. The second approach considers each task individually, using methods such as transfer learning or meta-learning to adapt policies across different tasks. For instance, some studies propose reward shaping based on demonstrations to guide policy transfer [29] or design auxiliary rewards to encourage leveraging policies from previous tasks in new contexts [30]. However, these methods predominantly focus on policy-based deep reinforcement learning and rely on experience data, including transitions and rewards. In contrast, our approach focuses on leveraging the value function. We theoretically demonstrate that the value function inherently encapsulates valuable information about the task distribution in sparse reward scenarios. We argue that this information alone is sufficient to transfer useful knowledge across tasks, eliminating the need for computing dense rewards [31].

**Tasks distributional information:** Furthermore, our research explores the role of distributional information in transfer reinforcement learning. Most studies addressing this issue are within the discipline of meta-reinforcement learning. For example, ref. [16] uses probabilistic distributions of latent task variables to facilitate transfer between tasks by inferring them from experience. Other works tackle the challenge of distribution shifts between offline meta-training and online meta-testing, proposing in-distribution adaptation methods based on the Bayesian adaptive framework [14,15]. Some researchers have implemented reward shaping by dynamically adjusting the reward structure according to task distributions [30,32]. In contrast, our focus is on transfer learning rather than meta-learning. We study the distributional information within the values of the value function instead of the distributions of trajectories or rewards, and we do not involve distinct training and testing phases. Although our work is centered on a tabular setting, it has the potential to be adapted for meta-learning in the future.

## 3. Preliminaries

**Markov Decision Processes** Reinforcement learning formalizes the interaction between an agent and its environment using Markov decision processes (MDPs). An MDP M=〈S,A,r,g,T,T,γ〉 is defined by a state space *S*, an action space *A*, a reward function r:S×A×S→R, and a transition function T:S×A×S→[0,1], which specifies the probability of transitioning to the next state given the current state and action. Additionally, a discount factor γ∈[0,1] is used to weigh the importance of future rewards. We added two elements to the standard definition of the MDP:

- The terminal state g∈S, which represents the goal the agent must achieve. This state is terminal, which means the agent cannot take further actions from there.
- **The most-likely-next-state function**, defined as (T:S×A→S), which is a function that maps a state action to the most likely state among all possible next states, such as
  $$T\left(s,a\right)=\arg\max_{s^{\prime }}T\left(s,a,s^{\prime }\right)$$
  For example, in a grid-world scenario, taking an action such as moving left might result in a high probability of transitioning to the adjacent left cell, which can be captured by T(s,a).

**Sparse Binary Reward:** Environments defined by a terminal goal are often modeled by using a uniform negative reward for all states, except for the goal state, where the agent receives a positive reward [25,26]. This reward model allows the agent to discover the shortest or most efficient path to the goal. In our work, we use a binary reward function as an alternative formulation, which serves the same purpose as the negative sparse reward by also driving the agent toward the shortest path. The reward function assigns a binary value $r(s,a,s^{\prime })=0$ if $s^{\prime }\neq g$ and $r\left(s,a,s^{\prime }\right)=r_{g}$ otherwise, and we choose $r_{g}$ [31]. This binary reward structure will be consistent with our theoretical framework without loss of generality, as this reward function can be scaled if a negative reward is preferred.

**Episodes and returns:** The agent engages with the MDP through a series of iterations referred to as episodes. During each episode, the agent takes sequential steps within the environment, starting from any initial state. At any given step *t*, the interaction unfolds a path as follows:

$$\tau =s_{t},a_{t},s_{t+1},a_{t+1},\ldots$$

where $s_{t+1}∼T\left(s,a,s_{t+1}\right)$. The return $G_{t}$ in a state *s* is defined as the discounted cumulative reward $r_{t}=r\left(s_{t},a_{t},a_{t+1}\right)$:

$$G_{t}=r_{0}+\gamma r_{1}+\gamma^{2}r_{2}+\ldots =\sum_{t=0}^{\infty }r_{t}\gamma^{t}$$

**Value Function:** The agent’s primary objective is to discover a policy function π that maximizes the expected return, as expressed by the state-action function, which is denoted by *Q*:

$$Q^{\pi }\left(s,a\right)=E^{\pi }\left[\sum_{t=0}^{\infty }r_{t}\gamma^{t}\right]$$

The optimal policy is denoted as $\pi^{*}$ and is the best policy, and its corresponding optimal function is $Q^{*}\left(s,a\right)=argmax_{\pi }Q^{\pi }\left(s,a\right)$, where $\pi^{*}\left(s\right)=argmax_{a}Q^{*}\left(s,a\right)$.

**Lifelong Learning:** Lifelong learning refers to a specific setting of transfer learning where the agent is assigned tasks continuously from a distribution—namely, Ω [2,10]. In RL, each task is equivalent to a distinct MDP, such as $M_{i}∼\Omega$. The main objective is to extract knowledge from a sample of past learned tasks $\left\{M_{0}..M_{N}\right\}$ to learn new tasks drawn from the same distribution $M_{N+1..\infty }∼\Omega$.

## 4. Expected Optimal Path Length

This section introduces the concept of “expected optimal path length” which technically captures the number of steps required for an agent to reach a goal state under a given policy. Here, we formally define this concept, derive its properties, and explore its implications in value function.

### 4.1. Definition and Representation of EOPL

Let’s consider a policy π, where an agent follows this policy starting from any initial state $s_{0}$ and reaches the terminal state goal (In the literature, sequences are typically defined as starting at a specific time *t* and ending at time *H*, with the length of the sequence being given by H−t. To simplify the notation for later theorems and propositions, we choose to refer to the state at time *t* as 0. This adjustment relaxes the notation while preserving generality), the generated path (sequence of states and actions) τ(π) is the path that generates from this policy and is represented as follows:

$$\tau \left(\pi \right)=s_{0},\pi \left(s_{0}\right),s_{1},\pi \left(s_{1}\right),\ldots ,s_{H-1},\pi \left(s_{H-1}\right),s_{H}\equiv g$$

The term *H* denotes the step of episode termination. The value of *H* can be a constant in the case of a finite horizon or an integer representing the length of the sequence if the termination condition reaches the goal state *g*, as in our case. In this scenario, the length of the sequence is the expected path length under the stochastic dynamics, expressed as follows:

$$H=E_{s_{t}∼T\left(s_{t-1},a_{t-1},s_{t}\right)}\left[\sum_{s_{t}=s_{1}}^{s_{t}=g}1|\forall s_{t}\in \tau \left(\pi \right)\right]$$

Intuitively, the optimal sequence of the expected path, given a policy π, consists of each successor state being the most likely one following the action prescribed by π, i.e., $s_{t+1}=T\left(s_{t},\pi \left(s_{t}\right)\right)$. This can be represented as follows:

$$\tau^{*}\left(\pi \right)=s_{0},\pi \left(s_{0}\right),T\left(s_{0},\pi \left(s_{0}\right)\right),a_{1},\ldots ,s_{H-1},a_{H-1},s_{H}\equiv g$$

since the next state is deterministic, we remove the expectation, and the length of $\tau^{*}\left(\pi \right)$ becomes

$$H=\left[\sum_{T_{t}=T_{1}}^{T_{t}=g}1|\forall s_{t}\in \tau^{*}\left(\pi \right)\right]\;\;;T_{t}=T\left(s_{t-1},\pi \left(s_{t-1}\right)\right)$$

From the latter equation, we deduce that the quantity *H* is dependent on the initial state and the policy, so we introduce a function that provides the number of steps given the policy π and the state *s*. Since we will be addressing the state-action function *Q* in the rest of the paper, we define this function as receiving two inputs—the state and action—and denote the number of steps as 1 for transitioning to the corresponding next state by taking action *a*, followed by the policy π.

**Definition 1** (Expected Optimal Path Length (EOPL))**.** *$ℋ:S\times A\to N_{0}$ is a function representing the EOPL given the state-action pair and the policy, with the following properties:*

*1.*  *${ℋ}^{\pi }\left(s,a\right)⩾1$ if s≠g else ${ℋ}^{\pi }\left(s,g\right)=0$.*
*2.*  *${ℋ}^{\pi }\left(s,a\right)=1+{ℋ}^{\pi }\left(s^{\prime },\pi \left(s^{\prime }\right)\right)$, where $s^{\prime }=T\left(s,a\right)$.*

The properties can be interpreted as follows:

- The EOPL is 1 if the most likely successor state following action *a* from state *s* is the goal *g*. The agent cannot take any further actions if it is already in the goal state *g*, as this state is terminal; thus, the optimal length is 0.
- For any state different from the goal state *g*, the EOPL is strictly greater than 0.

### 4.2. Value Function in Term of EOPL

We can draw some properties of the value function by considering the characteristics of the MDP in question. For instance, in an MDP with a sparse positive reward, where the optimal policy should output the shortest path to the goal, the EOPL of $\pi^{*}$ becomes the minimum policy length and influences the value function. Based on this, the value function expectation can be rewritten as follows:

**Proposition** **1.** Given ${ℋ}^{*}\left(s,a\right)$ as the EOPL of the optimal policy $\pi^{*}$, the expected value function can be formulated as follows:

$$Q^{*}\left(s,a\right)=E^{*}\left[\sum_{t=0}^{\infty }r_{t}\gamma^{t}\right]=E^{*}\left[\sum_{t=0}^{{ℋ}^{*}\left(s,a\right)}r_{t}\gamma^{t}\right].$$

This proposition is proven by showing that, in sparse positive reward settings, ${ℋ}^{*}$ is the minimum possible EOPL among all possible policies, and rewards beyond achieving the goal state yield a reward equal to zero; thus, the summation in the value function can be truncated at ${ℋ}^{*}$. Appendix A.1 contains the detailed proof.

## 5. Value Function Distribution in Terms of EOPL Distribution

In this section, we delve into the initialization of the value function, outlining both the theoretical foundations and the intuition behind our approach. We begin by introducing the concept of decomposing the task distribution into EOPL distributions and proceed to provide theoretical proofs for both reward and dynamics distribution scenarios by presenting the mathematical propositions that support our method.

### 5.1. Value Function Initialization by the Distribution Expectation

The initialization of the value function is a method of knowledge transfer where the agent is assigned its value function before engaging with the environment—namely, at episode ∅ [11], where the initialized value function is denoted as $Q^{\emptyset }$. The initialization is analogous to prediction, which means estimating the closest value possible based on the experiences acquired from past tasks. Hence, given the distribution Ω, we express the initialization process of the value function of the new task $M_{new}∼\Omega$ as the expectation from the value functions from the same distribution. Instead of assigning an entity of the value function from the data, such as $Q_{new}^{*}:=E_{M∼\Omega }\left[Q_{M}^{\emptyset }\right]$, we treat each state action separately and initialize each state action value of the new function:

$$\forall s,a\;Q_{new}^{\emptyset }\left(s,a\right):=E_{M∼\Omega }\left[Q^{*}\left(s,a\right)\right]$$

The most straightforward approach to calculating the expected value is to use the mean from the data of previously seen tasks [11]. While such a method proves effective, particularly for unknown distributions, we posit that the expectation could be more efficiently derived when the distribution is known. Hence, it is a key solution of the initialized function $Q^{\emptyset }\left(s,a\right)$.

### 5.2. Decomposing the Distribution of Similar State-Action Tasks

**Assumption** **1.** *If an agent is solving a set of tasks within the same state-action space, the primary distinction between the solutions for these tasks at any given state is the number of steps the agent takes to reach the goal.*

This assumption implies that, while the state-action spaces remain consistent across tasks, the differences in task solutions arise from variations in the agent’s learned policies and the optimal paths it discovers. Once an agent completes the learning process for a given task, it relies solely on the learned policy and no longer requires access to reward signals or the environment’s dynamics.

**Figure 1:**
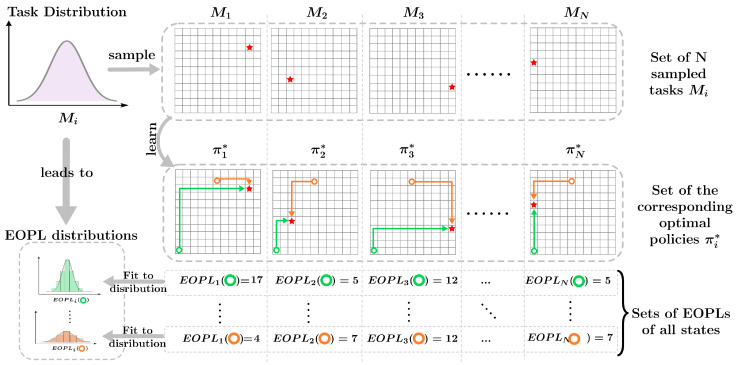
Illustration of the assumption of how task distributions can be decomposed into multiple distributions of expected optimal path lengths for all states.

For a set of tasks within the same state-action space, the agent can compute the EOPLs from any starting state to the goal for each task. By aggregating these EOPLs across tasks, the agent effectively builds a dataset that captures how far each state is from the goal under different task dynamics or reward structures. This dataset can then be fit into a distribution with its own parameters, representing the variation in EOPLs across tasks. We refer the reader to Figure 1, which provides an illustration of the assumption under discussion.

**Proposition** **2.** *Let Ω represent a distribution of tasks $\left\{M_{i}|M_{i}∼\Omega \right\}$, all sharing the same state-action space. Then, the expected value of the optimal value function over this task distribution can be expressed as follows:*

$$E_{M∼\Omega }\left[Q^{*}\right]\equiv \forall s,a\in S\times A,\;E_{M∼\Omega }\left[Q^{*}\left(s,a\right)\right]=E_{{ℋ}^{*}\left(s,a\right)∼ℌ\left(s,a\right)}\left[\sum_{t=0}^{{ℋ}_{i}\left(s,a\right)}r_{t}\gamma^{t}\right],$$

*where ℌ(s,a) represents the distribution of EOPL in (s,a).*

To validate this proposition, we independently analyze the case of goals distribution and dynamics distribution. In the following sections, we provide detailed derivations for these cases, demonstrating how the task distribution is composed of all the state actions’ EOPL distributions.

### 5.3. Goal Distribution

**Figure 2:**
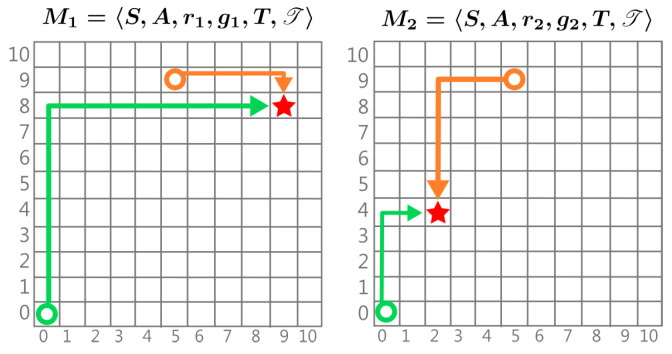
Example of two MDPs with different rewards. The circle represents the state, the arrows represent the EOPL, and the star represents the goal. In $M_{1}$ with the goal position g=[8,9], we observe EOPL(◯)=17, EOPL(◯)=4. In $M_{2}$, the goal position is g=[4,2]; therefore EOPL(◯)=5, EOPL(◯)=7.

The scenario of goal distributions (illustrated by Figure 2) is often related to tasks with the objective of path planning and finding the shortest path to achieve a dynamically changing goal [25,27]. The variation of the goals from one task to another implies the changes in reward. Hence, a task sampled from a distribution of goals $M_{i}∼\Omega_{g}$ is written as $M_{i}=\langle S,A,r_{i},g_{i},T,T,\gamma \rangle$.

**Proposition** **3.** *If $M∼\Omega_{g}$, then ∀(s,a) there exists a distribution ℌ(s,a) such as*

$$E_{M_{i}∼\Omega_{g}}\left[Q^{*}\left(s,a\right)\right]=E_{{ℋ}_{i}^{*}\left(s,a\right)∼ℌ\left(s,a\right)}E^{*}\left[\sum_{t=0}^{{ℋ}_{i}^{*}\left(s,a\right)}r_{t}\gamma^{t}\right]$$

**Proof.**  Suppose we have a distribution $\Omega_{g}$ where $\forall M_{i}\in \Omega_{g}\;\;M_{i}=\langle S,A,r_{i},g_{i},T,T\rangle$. Intuitively, the same state on the different tasks will be located at different distances from the goal; therefore, if we have

**Lemma** **1.** If $M_{i}$ and $M_{j}$ are two tasks from the distribution Ω where $g_{i}\neq g_{j}$, then there exists at least one s,a where ${ℋ}_{i}^{*}\left(s,a\right)\neq {ℋ}_{j}^{*}\left(s,a\right)$

**Proof.**  According to the properties of ℋ, we have ${ℋ}_{i}^{*}\left(g_{i},a\right)=0$, since $g_{i}$ is terminal and the agent cannot take any further actions. On the other hand, we have ${ℋ}_{j}^{*}\left(g_{i},a\right)>0$, since $g_{i}\neq g_{j}$, and therefore, $g_{i}$ is not terminal in $M_{j}$, and the distance between the state $g_{i}$ in $M_{j}$ and its goal $g_{j}$ is non-zero. Consequently, we have ${ℋ}_{i}^{*}\left(g_{i},a\right)\neq {ℋ}_{j}^{*}\left(g_{i},a\right)$, establishing the validity of the proposition.     □

Therefore, we can extract the set of ${ℋ}_{i}^{*}$:

$$ℌ\left(s,a\right)=\left\{{ℋ}_{i}^{*}\left(s,a\right),\forall M_{i}\in \Omega_{g}\right\}$$

For any new task from the distribution, the expectation of its value function can be reformulated in terms of its horizon distribution:

$$E_{M∼\Omega_{g}}\left[Q^{*}\left(s,a\right)\right]=E_{M∼\Omega_{g}}E^{*}\left[\sum_{t=0}^{{ℋ}^{*}\left(s,a\right)}r_{t}\gamma^{t}\right]=E_{{ℋ}^{*}\left(s,a\right)∼ℌ\left(s,a\right)}E^{*}\left[\sum_{t=0}^{{ℋ}^{*}\left(s,a\right)}r_{t}\gamma^{t}\right]$$

This establishes that the expectation of the value function $Q^{*}\left(s,a\right)$ is dependent on the EOPL distribution ${ℋ}^{*}$ sampled from the tasks distribution $\Omega_{g}$.     □

### 5.4. Dynamics Distribution

**Figure 3:**
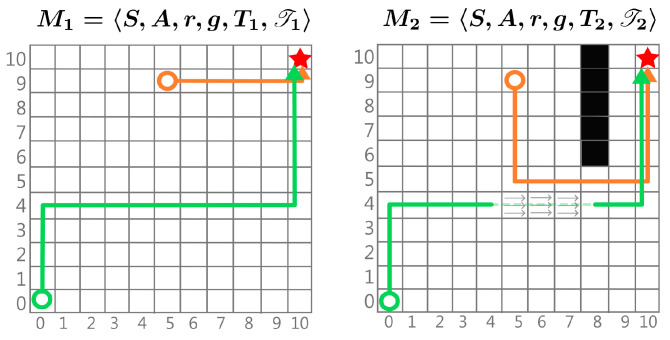
Example illustrating two MDPs with different dynamics. A circle represents a state, the arrows are the EOPL, and the star represents the goal. Here, we observe EOPL(◯)=19 and EOPL(◯)=6. In $M_{2}$, new dynamics are introduced, such as the transition from state [6,8] to [10,8], representing an impassable wall, which results in a longer EOPL of EOPL(◯)=15 compared to $M_{1}$. Additionally, in the transition from [4,5] to [4,7], a pushing force moves the agent three states to the right, reducing EOPL(◯)=16 and making it shorter than in $M_{1}$.

Environment dynamics refer to the rules that govern how the agent moves to the next state in response to the actions taken in the environment. Formally, in an MDP, the dynamics function *T* determines the probabilities of transitioning to all states in the environment after taking action *a* from any state *s*. This function assigns probabilities mainly to neighbouring states that can be directly accessed by available actions, with all other states having probabilities of 0 since they are inaccessible from that state. Changes in dynamics within tasks sharing the same state and action space can be categorized into two scenarios (which are depicted in the illustrative example in Figure 3):

1. **Different *T* Similar T:** Only the probability of transitioning to the same neighbouring state using the same action changes slightly. In this case, T(s,a) remains nearly the same, so the EOPL does not significantly change across tasks.
2. **Different *T* Different T:** The same state-action pair may result in transitions to different states in different tasks. In this case, the EOPL changes depending on whether the transition moves the agent closer to the target or farther away. For example, consider a drone in a windy environment [9], where the target is always the same. Due to varying wind conditions, the drone will take different paths to reach the target each time. If the wind pushes it towards the target, it will reach the target more quickly; if the wind pushes against it, the drone will take longer to achieve the goal.

Let $\Omega_{T}$ be a distribution of tasks with different transitions such as $\forall M_{i}\in \Omega_{T},\;M_{i}=\langle S,A,r,g,T_{i},T_{i},\rangle$. We consider *g* to be unchanged over the tasks but without loss of generality, as this assumption helps prove mathematically that the change in dynamics is a change that can be translated as a change in EOPL.

**Proposition** **4.** *If $M∼\Omega_{T}$, ∀(s,a), there exists a distribution ℌ(s,a) such that*

$$E_{M∼\Omega_{T}}\left[Q^{*}\left(s,a\right)\right]=E_{ℋ(s,a)∼ℌ(s,a)}E^{*}\left[\sum_{t=0}^{ℋ(s,a)}r_{t}\gamma^{t}\right]$$

**Proof.**  Unlike the proposition concerning goal distribution, we cannot assert with certainty that each ${ℋ}_{i}$ is unique, as this depends on the degree of variance in dynamics across tasks and their similarities. Therefore, we limit our claim to demonstrating that each task certainly possesses a unique ${ℋ}_{i}$ due to the lack of conclusive evidence to the contrary, as outlined in the following lemma.

**Lemma** **2.** *For $M_{i}$ and $M_{j}$, as two tasks from the distribution $\Omega_{T}$ where $g_{i}=g_{j}$ and $T_{i}\neq T_{j}$, each $M_{i}\in \Omega_{T}$ has its unique ℋ.*

**Proof of Lemma** **2.** Since $T_{i}\neq T_{j}$, there exists at least one s,a such that $T_{i}\left(s,a\right)\neq T_{j}\left(s,a\right)$. Therefore, using Property 4 of the following definition:

$${ℋ}_{i}\left(s,a\right)=1+{ℋ}_{i}\left(T_{i}\left(s,a\right),\pi_{i}\left(T_{i}\left(s,a\right)\right)\right)$$

$${ℋ}_{j}\left(s,a\right)=1+{ℋ}_{j}\left(T_{j}\left(s,a\right),\pi \left(T_{j}\left(s,a\right)\right)\right)$$

Hence, the relationship between ${ℋ}_{i}\left(s,a\right)$ and ${ℋ}_{j}\left(s,a\right)$ depends on whether ${ℋ}_{j}\left(T_{j}\left(s,a\right),\pi \left(T_{j}\left(s,a\right)\right)\right)$ equals ${ℋ}_{i}\left(T_{i}\left(s,a\right),\pi_{i}\left(T_{i}\left(s,a\right)\right)\right)$. Due to insufficient information, we cannot definitively state their equality; thus, ${ℋ}_{i}\left(s,a\right)⪋{ℋ}_{j}\left(s,a\right)$.

In the edge case scenario, if $\forall M_{i},M_{j}\in \Omega_{T},T_{i}\left(s,a\right)\neq T_{j}\left(s,a\right)$ but ${ℋ}_{i}\left(s,a\right)={ℋ}_{j}\left(s,a\right)$, it implies that the distribution of tasks has negligible variance and is not worth investigating. However, if the distribution of dynamics is sufficiently large to create significant variability between tasks, then ${ℋ}_{i}\left(s,a\right)⪋{ℋ}_{j}\left(s,a\right)$ holds, and therefore, each $M\in \Omega_{T}$ has its unique ℋ.     □

Based on this lemma, we deduce that ℌ(s,a) is a distribution such that

$$ℌ\left(s,a\right)=\{{ℋ}_{i}\left(s,a\right)|\forall M_{i}\in \Omega_{T}\}$$

Therefore, we can write

$$\begin{array}{cc} E_{M∼\Omega_{T}}\left[Q^{*}\left(s,a\right)\right] & =E_{M∼\Omega_{T}}E^{*}\left[\sum_{t=0}^{{ℋ}^{*}\left(s,a\right)}r_{t}\gamma^{t}\right]\\ \; & =E_{ℋ(s,a)∼ℌ(s,a)}E^{*}\left[\sum_{t=0}^{{ℋ}^{*}\left(s,a\right)}r_{t}\gamma^{t}\right] \end{array}$$

This establishes that the expectation of the value function $Q^{*}\left(s,a\right)$ depends on the horizon distribution ℋ sampled from the tasks distribution $\Omega_{T}$.    □

### 5.5. Value Function Distribution

We have established that the distribution of an MDP can be expressed in terms of the distribution of the path lengths generated by their policies. However, for transfer learning, our focus shifts to the distribution of the value function, as the agent will have access to the value function as part of the learning process, rather than the trajectory lengths. To formalize this, we define Q as the distribution of the value functions for all tasks within the distribution $\Omega \in \{\Omega_{g},\Omega_{T}\}$. In this context, $Q=\{Q_{i}|M_{i}\in \Omega \}$.

Consequently, the distribution of the value function can be represented as follows:

$$Q\left(s,a\right)=\left\{E^{*}\left[\sum_{t=0}^{{ℋ}_{i}^{*}\left(s,a\right)}r_{t}\gamma^{t}\right]\;|\;{ℋ}_{i}^{*}\left(s,a\right)\in ℌ\left(s,a\right)\right\}$$

While the general results apply to all MDPs, we now shift our attention to SBR-MDPs, which are characterized by binary reward structures.

**Proposition** **5.** Given MDP $M_{i}$, where g is a terminal state and the reward function is a positive binary reward, using the Bellman equation [1] on the expected optimal path of the optimal policy $\pi^{*}$, the state-action value function is as follows:

(1) $$Q^{*}\left(s,a\right)=\gamma^{{ℋ}^{*}\left(s,a\right)}\prod_{t=0}^{{ℋ}^{*}\left(s,a\right)}T\left(s_{t},a_{t},s_{t+1}\right)$$

**Proof.**  See Appendix A.2.     □

Thus, the distribution of the value function can be written as follows:

$$Q\left(s,a\right)=\left\{\gamma^{{ℋ}_{i}^{*}\left(s,a\right)}\prod_{t=0}^{{{ℋ}_{i}}^{*}\left(s,a\right)}T\left(s_{t},a_{t},s_{t+1}\right)|{ℋ}_{i}^{*}\left(s,a\right)\in ℌ\left(s,a\right)\right\}.$$

## 6. Case Study: Log-Normality of the Value Function Distribution

To test the value function distribution and its connection to EOPL distributions, we test the case where EOPL follows a normal distribution. In RL, the normal distribution often emerges due to its presence in nature and its ability to formulate various random events and uncertainties within the task elements [33]. For example, many algorithms assume that rewards, transition dynamics or noise in the environment follow a Gaussian distribution, as it effectively models real-world variability [7]. This makes the normal distribution a natural fit for representing the EOPL.

**Proposition** **6.** *If $\forall \left(s,a\right)\;\;ℌ\left(s,a\right)\equiv N\left(\mu_{ℌ(s,a)},\sigma_{ℌ(s,a)}^{2}\right)$, then the value function distribution Q(s,a) follows a log-normal distribution such as*

$$\ln Q\left(s,a\right)∼N(\mu_{Q}\left(s,a\right),\sigma_{Q}^{2}\left(s,a\right))$$

**Proof.**  We apply the natural logarithm on both sides of Equation (1)

$$\begin{array}{cc} \ln Q(s,a) & ={ℋ}^{*}\left(s,a\right)\ln\gamma +\ln\left(\prod_{t=0}^{{ℋ}^{*}\left(s,a\right)}T_{t}\right)\\ \; & ={ℋ}^{*}\left(s,a\right)\ln\gamma +\sum_{t=0}^{{ℋ}^{*}\left(s,a\right)}\ln T_{t} \end{array}$$

If we assume ${ℌ}^{*}\left(s,a\right)\equiv N\left(\mu ,\sigma^{2}\right)$, then we can assume under certain conditions that $\sum_{t=0}^{{ℋ}^{*}\left(s,a\right)}\ln T_{t}$ follows a normal distribution using the central limit theorem. For the full proof, see Appendix A.3. Finally, we write

$$\ln Q\left(s,a\right)∼N(\mu_{Q}\left(s,a\right),\sigma_{Q}^{2}\left(s,a\right))$$

where the parameters $\mu_{Q}\left(s,a\right)$ and $\sigma_{Q}^{2}\left(s,a\right)$ depend on the scaling. Consequently, Q(s,a) follows a log-normal distribution.     □

### Algorithm: *LogQInit*

The log-normal distribution properties suggest two natural expectation strategies—mean and median. Accordingly, we introduce two LogQInit approaches as follows:

1. LogQInit-Median: The median, being robust to skewness, is calculated as follows:

$$Q^{\emptyset }\left(s,a\right):=e^{\mu (s,a)}$$

2. LogQInit-Mean: The mean, which reflects the average expected value, is given by

$$Q^{\emptyset }\left(s,a\right):=e^{\mu \left(s,a\right)+\frac{1}{2}\sigma^{2}\left(s,a\right)}$$

Algorithm 1 outlines the LogQInit method, which proceeds as follows:

- The agent first samples a subset of tasks which are learnt sufficiently to obtain a close approximation of their optimal function $Q^{*}$ and are stored in Q.
- the EOPL distribution parameters for each state action $\left(\mu_{Q}\left(s,a\right),\sigma_{Q}^{2}\left(s,a\right)\right)$ are then estimated using the sample.
- Using these estimates, the value function is initialized and learnt afterwards for subsequent tasks.

**Algorithm 1:** LogQInit**variables:** Ω; σ:S×A; μ:S×A;n:numberoftrainingtasks**for** i=1 **to** *n* **do**    sample new task $M_{i}∼\Omega$    ${\mathrm{learnQ}}^{*}\left(M_{i}\right)$    $Q\cup Q_{i}^{*}$**end for*****Estimate σ and μ*****for all** state-actions s,a **do**    $\sigma^{2}\left(s,a\right)\leftarrow Variance\left(\ln Q\left(s,a\right)\right)$    μ(s,a)←Mean(lnQ(s,a))**end for*****Starting lifelong learning*****repeat**    sample new task $M_{new}∼\Omega$    initialization: $Q_{new}^{\emptyset }\leftarrow e^{\mu (s,a)}$(median) or $e^{\mu \left(s,a\right)+\frac{\sigma^{2}\left(s,a\right)}{2}}$(mean)    learn**until** Agent lifetime

## 7. Experiments

The objectives of our experiments are summarized in two key points: Firstly, we aim to demonstrate that the distribution of tasks can be translated into distinct distributions of optimal paths for every state-action value, both in cases of goal distributions and of dynamics distributions. Secondly, we seek to test our hypothesis that, in the case of a normal task distribution, using the log-normal parameters for initialization (LogQInit) is more effective than existing initialization methods. To evaluate this, we compare LogQInit with the following strategies:

- MaxQInit [10]: A method based on the optimistic initialization concept, where each state-action value is initialized using the maximum value from previously solved tasks.
- UCOI [11]: This method balances optimism in the face of uncertainty by initializing with the maximum value while relying on the mean when certainty is higher.

We also compare with the following other popular transfer methods:

- PTRS (Policy Transfer with Reward Shaping) [17]: This approach utilizes the value function from the source task as a potential function to shape the reward in the target task, expressed as $r^{\prime }\left(s,a\right)=r\left(s,a\right)+\gamma \phi \left(s^{\prime }\right)-\phi \left(s\right)$. Here, the potential function is defined as the mean of the set Q.
- OPS-TL [34]: This method maintains a dictionary of previously learned policies and employs a probabilistic selection mechanism to reuse these policies for new tasks.
- DRS (Distance-Based Reward Shaping): We introduce this algorithm as a straightforward reward-shaping method that relies on the distance between the current state and the goal state.

Additionally, we compare these approaches with $Q^{*}$, which we claim we sufficiently obtained through Q-learning, and a non-transfer baseline, where the Q-function was initialized by zeros.

### 7.1. Goal Distribution: Gridworld

The first experiment investigates the normal distribution of goals in the standard Gridworld environment, which is commonly employed in RL theories [1]. Gridworld is a 2D map of states that is identified by its coordinates [i,j], enabling straightforward determination of the EOPL, which is typically quantified by the Manhattan distance from any state to the goal. Such a setting offers simplicity and efficacy in demonstrating theoretical propositions and visualizing the different experiment results.

#### 7.1.1. Environment Description

The environment is a 30×30 Gridworld, where each state is identified by its coordinates [i,j]. The agent can execute four actions (up, down, left, and right) across all states. When the agent takes an action, it transitions to the next state with a probability of $p=\max_{s^{\prime }}T\left(s,a,s^{\prime }\right)∼uniform\left(0.6,0.9\right)$, while other states have the probability of $\frac{1-p}{3}$. A reward of 1 is assigned to the terminal state, denoted as *g*, while all other states in the environment yield a reward of 0. Tasks are generated with diverse goals defined as $g=[i∼N\left(20,\sigma^{2}\right),j∼N\left(20,\sigma^{2}\right)]$. Various distributions are tested based on different variance values, specifically $\sigma^{2}\in \left[1,3,5,7,10\right]$, besides a uniform distribution where $g\left[i∼uniform(0,30),j∼uniform(0,30)\right]$. The goal state’s normal distribution leads with a normal distribution of distances from each state to the goal, and therefore, the normality of the EOPL’s distribution.

#### 7.1.2. Results and Discussion

**Figure 4:**
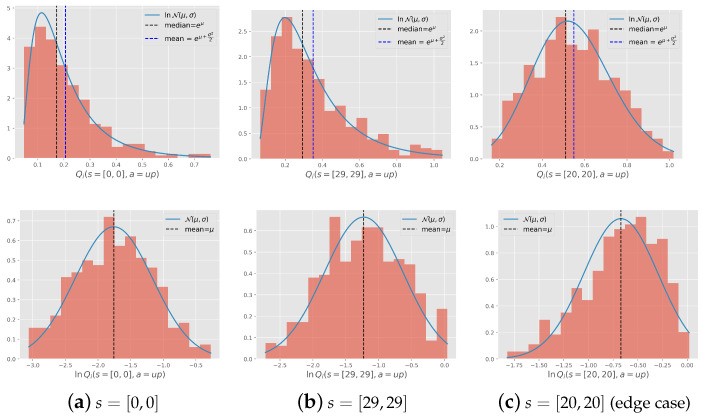
(**Top Row**) Histogram of Q(s,a) for 100 Gridworld tasks learned to completion; (**Bottom Row**) Corresponding histogram of lnQ(s,a). Each column corresponds to a state: (**a**) the state s=[0,0], (**b**) the state s=[29,29], (**c**) the state s=[20,20] which represents the edge case.

**Log-normality of the value function**: We initially trained the agent on 100 tasks drawn from the given distribution. These tasks were learned from scratch and assumed to be learned until completion. Figure 4 illustrates the histogram of a set of Q(s,a) values of the states s={[0,0],[20,20],[29,29]} obtained from learning these 100 tasks drawn from the distribution with the variance $\sigma^{2}=7$. By examining the histograms of s=[0,0] and s=[29,29] (Figure 4d and Figure 4e, respectively), we observed that the normal distribution of goals indeed resulted in a log-normal distribution of Q(s,a) with negligible divergence, likely stemming from the function approximation. The same did not apply to s=[20,20], where the distribution of Q(s,a) is itself normal since the goal is centered on this state. However, we argue that the choice of initialization values will not significantly impact learning. This is because the assigned value is high enough to prompt the agent to explore each state in this area, and the goal is likely to appear there, ensuring that exploration is not wasted.

**Figure 5:**
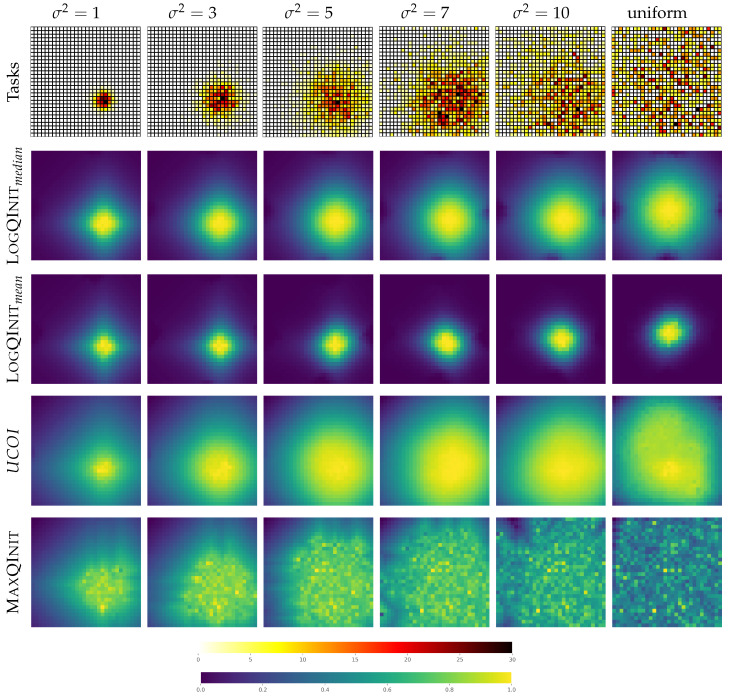
Heatmaps of the initial value functions in the Gridworld environment under various distribution scenarios.

**LogQInit** **vs. initialization methods:** The second phase of our analysis involves a comprehensive comparison between our proposed approach and existing methodologies. To begin, we visualize the initialized function and heatmaps, as depicted in Figure 5. Each column in the figure corresponds to a specific distribution with different variance, with the last column representing the uniform distribution. The first row represents the heatmap of goal distribution in the Gridworld environment, and the other rows each correspond to an initialization method.

Our findings reveal that our approach, LogQInit, generates a heatmap closely aligned with the task distribution. In essence, high values are centered around the center of the goal distribution, and the area of high values gradually expands with the increase in variance in the distribution. On the contrary, UCOI, though similar to LogQInit in assigning high values to areas with frequent goal appearances, exhibits a wider distribution of high values in scenarios with high variance. This implies that the agent may end up exploring more than it does with LogQInit. On the other hand, as previously demonstrated by [11], MaxQInit showcases an irregular pattern in the initial value function. While the heatmap resembles other approaches for $\sigma^{2}=1$, it becomes increasingly random and chaotic as $\sigma^{2}$ increases. This is attributed to two factors: MaxQInit follows an optimistic initialization that assigns the maximum value, and the sample size used is only 100. In contrast, the condition for MaxQInit requires using the probability of the least appearing MDP to compute the sample size, which results in needing to learn 767 tasks from scratch—a process that is both impractical and computationally intensive.

**Figure 6:**
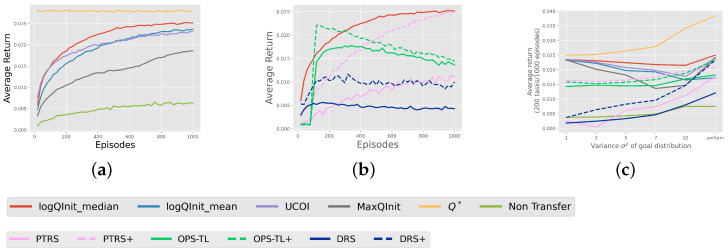
(**a**) average reward of initialization methods; (**b**) average reward of LogQInit vs. other transfer methods; (**c**) average reward against variance increase in task distribution. Average reward from transfer methods in Gridworld: (**a**) —average reward of initialization methods for the distribution of goals with $\sigma^{2}=7$; (**b**)—LogQInit-median compared with other transfer methods for the distribution of goals with $\sigma^{2}=7$; (**c**)—average reward over 100 episodes across different distributions.

The results deduced from the heatmaps are backed up by the comparison of average rewards obtained from learning 200 tasks using these approaches, as depicted in Figure 6. It is evident that LogQInit outperforms other approaches for all distributions, except when $\sigma^{2}=1$, since the initial value functions are basically similar. LogQInit-mean ranks third due to its lack of allowance for exploration, and MaxQInit yields the least favourable results among the compared approaches.

**LogQInit** **vs. transfer methods:** Figure 6b shows a comparison between LogQInit-median and other transfer methods—PTRS OPS-TL, and DRS—where + besides the latter methods indicates that the value function was initialized by LogQInit-median, and the absence of + indicates that it was initialized by zero. Notably, LogQInit shows better performance.

### 7.2. Dynamics Distribution: Mountain Car

The second experiments are directed for dynamics distribution in the environment. We chose the mountain car, a classic control problem introduced in the field of reinforcement learning [35].

#### 7.2.1. Environment Description

**Figure A1:**
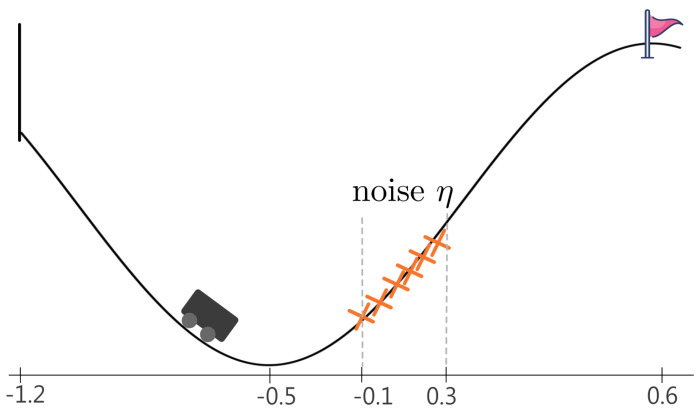
Mountain Car environment with added noise on the hill.

The car is situated in a valley between two hills, with the objective of reaching a flag positioned at the peak of one of the hills. The state space of the mountain car environment is defined by the car’s position ∈[−1.2,0.6] and velocity ∈[−0.07,0.07]. The action space is represented as A={0,1,2}, where 0 corresponds to doing nothing, 1 represents accelerating forward, and 2 represents decelerating or accelerating backwards. To illustrate a distribution of dynamics, we add a normal distribution of noise to the environment, particularly in the right hill area, as depicted in Figure A1. The dynamics are modeled as $position_{t+1}=position_{t}+velocity_{t}+\eta$, where $\eta ∼N(0,\sigma^{2})$. The variance $\sigma^{2}$, takes different values in various distribution scenarios, such as $\sigma^{2}\in \left[0.0002,0.005,0.01,0.015,0.02\right]$.

#### 7.2.2. Results and Discussion

**Figure 7:**
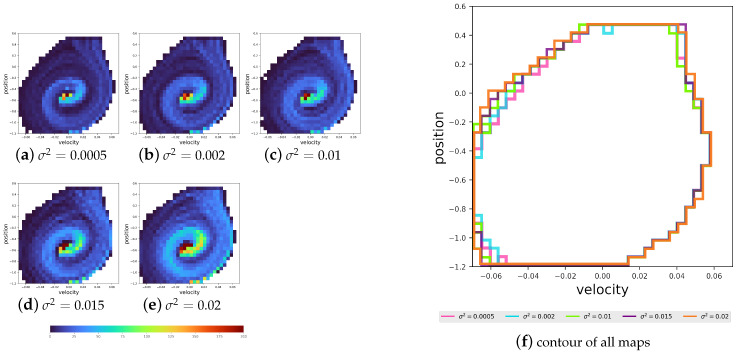
Visualization of all the states’ occurrences in the EOPL across 100 tasks. The x-axis represents velocity, and the y-axis represents position. **Left**: heatmaps showing the occurrence frequency for each distribution. **Right**: a close-up view of the heatmaps’ outlines for all the distributions.

**Figure 8:**
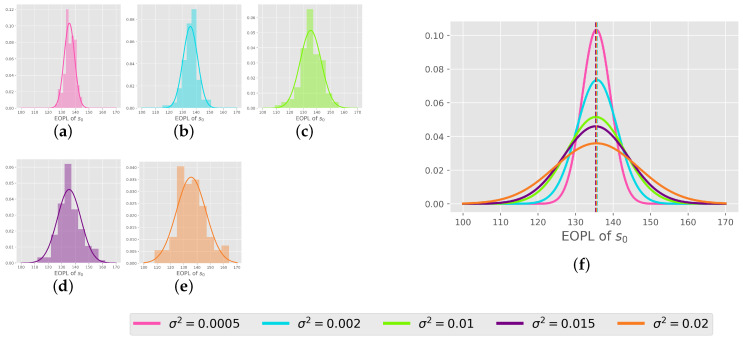
(**a**) $\sigma^{2}=0.0005$; (**b**) $\sigma^{2}=0.002$; (**c**) $\sigma^{2}=0.01$; (**d**) $\sigma^{2}=0.015$; (**e**) $\sigma^{2}=0.02$; (**f**) distribution functions of the EOPLs for all different distributions of noise. Histogram of the EOPLs of the state $s_{0}=\left[p=-0.5,v=0\right]$ across the 100 tasks. **Left**: histogram of each distribution. **Right**: plot of all density functions of all the distributions of noise together.

**Normality of the EOPL distribution:** We initiated the experiment by learning 100 tasks independently for each distribution without transfer for $5\times 10^{5}$ episodes using tabular Q-learning (details are provided in Appendix A.4). Firstly, we want to check the EOPL resulting from the 100 tasks and whether it abides by the Proposition 4. Therefore, we test the final policy starting from state $s_{0}$ tasks. We counted the occurrences of all the possible states within the path generated by the policy. These counts are in the shape of a grid tuple [position,velocity], which is visualized as a heatmap in Figure 7. Notably, certain states manifest more frequently in distributions with larger variances compared to those with smaller variances. This observation indicates that changes in this kind of dynamics lead to the different frequency of some states due to the difference in their function T, as do the sequences of states in the EOPL across tasks. Next, to prove that the EOPLs also differ in length, we displayed the EOPLs from the 100 tasks as histograms accompanied by their corresponding density functions, as shown in Figure 8. Remarkably, we observe that the distribution of the number of steps closely adheres to a normal distribution, aligning with our initial hypothesis. To compare the various distributions, we present their density functions in a unified plot, as illustrated in Figure 8. Notably, all distributions exhibit nearly identical means, corresponding to the number of steps obtained in an environment without noise. Moreover, we observe that the larger the variance in the distribution, the wider the variance in the EOPL distribution.

**Figure 9:**
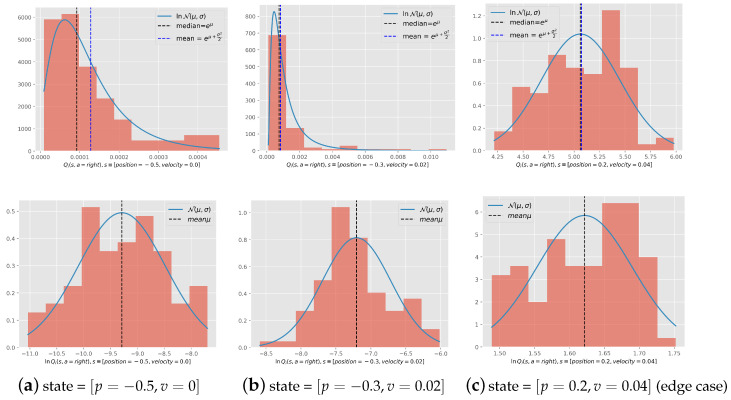
Histogram of Q(s,a) of 100 tasks ran into completion (**top row**) and its correspondent set of lnQ(s,a) (**lower row**) for the states. [p,v] is the abbreviation for [position,velocity].

**log-normality of the value function:** We have displayed the histogram of the value function in Figure 9 for the states [position = −0.5, velocity = 0.0] corresponding to the starting state, [position = −0.3, velocity = 0.02] corresponding to a state in the middle of the path, and [position = 0.2, velocity = 0.04] corresponding to a state that reaches the target. We notice that the histogram of 100 tasks of these functions follows a log distribution and a normal distribution just as hypothesized, except for the third state, which corresponds to the edge case explained in Appendix A.3.

**Figure 10:**
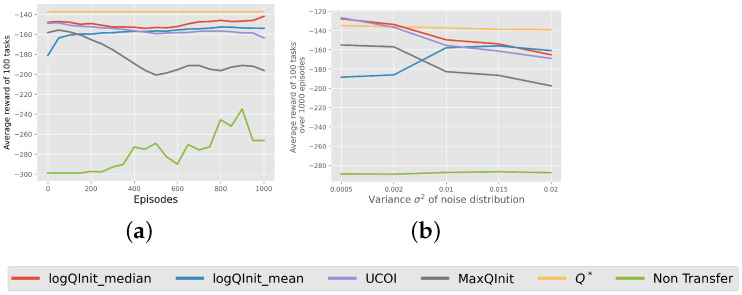
(**a**) average reward of distribution $sigma^{2}=0.01$; (**b**) average reward of each distribution of noise. Average reward of 100 tasks for the mountain car environment using different transfer methods.

LogQInit Performance: Finally, Figure 10 presents the average return obtained using different initialization methods. The results indicate that LogQInit-median, followed by UCOI, achieved the best performance in environments with lower variance in the noise distribution. In contrast, LogQInit-mean performed better in higher-variance settings, likely due to the need for more cautious exploration in such environments. However, we did not include a comparison with other transfer methods, as they provided no measurable improvement over 1000 episodes.

## 8. Conclusions

In this paper, we examined the distribution of state-action value functions induced by a distribution of tasks. Specifically, we studied tasks that had the same state-action space but differed in dynamics or rewards. We demonstrated that the value functions of these MDPs can be directly expressed in terms of state action-specific finite horizons by extracting the expected optimal path length (EOPL) of each state-action pair. This formulation established a direct connection between the task distribution and the resulting distribution of the value function.

We focused on MDPs with a terminal goal and sparse positive rewards, as this setting is widely used in the literature. This MDP configuration revealed an exponential relationship between the value function and EOPLs.

To validate our propositions, we examined the case where the task distribution follows a normal distribution. We showed that this results in a log-normal distribution of value functions, leading to the proposal of **LogQInit**, an initialization method based on the median and mean of the value function distribution. We tested this approach on task distributions where the resulting EOPLs are normally distributed. The results confirmed our hypothesis and showed that **LogQInit** provides a more accurate initialization than existing methods.

However, our work has two main limitations. First, we only tested normal and uniform distributions. In real-world applications, the distribution of optimal path lengths may be more complex, especially in environments where the dynamics function does not transition linearly across states. Extending this study to other types of distributions could further improve our approach. Second, our initialization method was evaluated in a tabular setting. A potential improvement is to discretize the state-action space and adapt the initialization process to continuous state spaces.

## Figures and Tables

**Figure A2:**
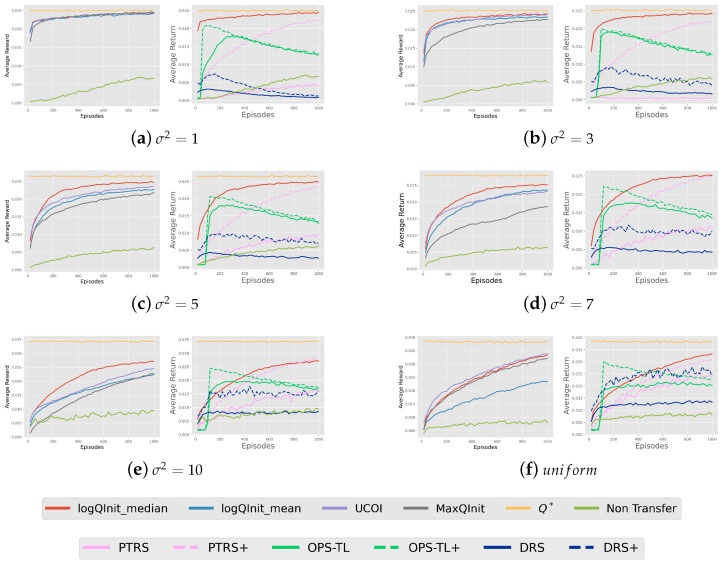
average return of on Gridworld using the different initialization methods and transfer methods.

**Figure A3:**
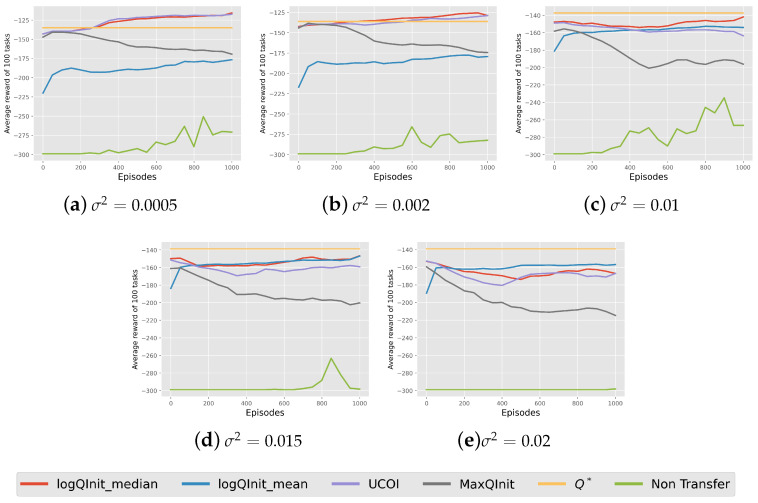
average return of the different initialization methods performance in mountain car environment across different noise distribution.

## Data Availability

Data is contained within the article.

## Appendix A

### Appendix A.1. Proof of Proposition 1

**Proof.** To prove that in an MDP with a terminal goal and positive rewards, the value function can be expressed as:

$$E\left[\sum_{t=0}^{\infty }\gamma^{t}r\left(s_{t},a_{t}\right)\right]=E\left[\sum_{t=0}^{{ℋ}^{*}\left(s,a\right)}r_{t}\gamma^{t}\right]$$

we need to show that ${ℋ}^{*}\left(s,a\right)=\min_{\pi }{ℋ}^{\pi }\left(s,a\right)$, which implies:

$$\forall \pi ,\;E\left[\sum_{t=0}^{{ℋ}^{*}\left(s,a\right)}r_{t}\gamma^{t}\right]\geq E\left[\sum_{t=0}^{{ℋ}^{\pi }\left(s,a\right)}r_{t}\gamma^{t}\right].$$

Assume there exists a policy $\pi^{+}$ such that ${ℋ}^{\pi^{+}}\left(s,a\right)<{ℋ}^{\pi^{*}}\left(s,a\right)$. Since the goal state *g* is terminal, no rewards are collected beyond reaching *g*. Therefore, for t>ℋ(s,a), we have $r(s_{t},a_{t})=0$. This allows us to rewrite the infinite-horizon expectation as:

$$E^{*}\left[\sum_{t=0}^{\infty }\gamma^{t}r\left(s_{t},a_{t}\right)\right]=E^{*}\left[\sum_{t=0}^{{ℋ}^{*}\left(s,a\right)}\gamma^{t}r\left(s_{t},a_{t}\right)+\sum_{t={ℋ}^{*}\left(s,a\right)+1}^{\infty }\gamma^{t}\cdot 0\right]$$

and

$$E^{+}\left[\sum_{t=0}^{\infty }\gamma^{t}r\left(s_{t},a_{t}\right)\right]=E^{+}\left[\sum_{t=0}^{{ℋ}^{\pi^{+}}\left(s,a\right)}\gamma^{t}r\left(s_{t},a_{t}\right)+\sum_{t={ℋ}^{\pi^{+}}\left(s,a\right)+1}^{\infty }\gamma^{t}\cdot 0\right].$$

Since ${ℋ}^{\pi^{+}}\left(s,a\right)<{ℋ}^{\pi^{*}}\left(s,a\right)$, it follows that:

$$E^{\pi^{+}}\left[\sum_{t=0}^{{ℋ}^{\pi^{+}}\left(s,a\right)}r_{t}\gamma^{t}\right]>E^{\pi^{*}}\left[\sum_{t=0}^{{ℋ}^{\pi^{*}}\left(s,a\right)}r_{t}\gamma^{t}\right].$$

This contradicts the definition of $Q^{*}$, which states that the optimal policy maximizes the expected return. Hence, we conclude that:

$${ℋ}^{*}\left(s,a\right)=\min_{\pi }{ℋ}^{\pi }\left(s,a\right).$$

□

### Appendix A.2. Proof of Proposition 5

**Proof.** To prove Propostion 5, we use the bellman optimality equation since we are dealing with the optimal function $Q^{*}$ which is consistent:

$$Q^{*}\left(s,a\right)=r\left(s,a,s^{\prime }\right)+\gamma T\left(s,a,s^{\prime }\right)\max_{a^{\prime }}Q^{*}\left(s^{\prime },a^{\prime }\right)$$

We set that s,a on the left side of the equation to be starting from time t=0. the EOPL of the state $s_{0}$ and action $a_{0}$, with $s_{t+1}=T\left(s_{t},\pi \left(s_{t}\right)\right)$ is:

$$\tau^{*}\left(\pi^{*}\right)=s_{0},a_{0},s_{1},\pi^{*}\left(s_{1}\right),\ldots ,s_{{ℋ}^{*}\left(s_{0},a_{0}\right)-1},\pi^{*}\left(s_{{ℋ}^{*}\left(s_{0},a_{0}\right)-1}\right),g$$

By definition we have $\pi^{*}\left(s\right)=argmax_{a}Q^{*}\left(s,a\right)$ therefore $max_{a}Q^{*}\left(s,a\right)=Q^{*}\left(s,\pi^{*}\left(s\right)\right)$ we run the bellman equation on the sequence $\tau^{*}\left(\pi^{*}\right)$, specifying that $r_{t}=r\left(s_{t},a_{t},s_{t+1}\right)$ and $T_{t}=T\left(s_{t},a_{t},s_{t+1}\right)$

$$\begin{array}{cc} Q^{*}\left(s_{0},a_{0}\right) & =r_{0}+\gamma T_{0}Q^{*}\left(s_{1},\pi^{*}\left(s_{1}\right)\right)\\ \; & =r_{0}+\gamma T_{0}\left(r_{1}+\gamma T_{1}\left(r_{2}+\gamma T_{2}\left(\ldots \left(r_{{ℋ}^{*}\left(s_{0},a_{0}\right)}+\gamma T_{{ℋ}^{*}\left(s_{0},a_{0}\right)-1}r_{g}\right)\ldots \right)\right)\right)\\ \; & =\gamma T_{0}\left(\gamma T_{1}\left(\gamma T_{2}\left(\ldots \left(\gamma T_{{ℋ}^{*}\left(s_{0},a_{0}\right)}\right)\ldots \right)\right)\right)\mathrm{by}\;\mathrm{substituing}\;\mathrm{positive}\;\mathrm{sparse}\;\mathrm{reward}\\ \; & =\gamma^{{ℋ}^{*}\left(s_{0},a_{0}\right)}\prod_{t=0}^{{ℋ}^{*}\left(s_{0},a_{0}\right)}T_{t} \end{array}$$

□

### Appendix A.3. Proof of Normality of $\sum_{t=0}^{ℋ(s,a)}\ln T_{t}$

**Proof.** We consider the transitions $T_{1},T_{2},\ldots ,T_{ℋ(s,a)}$ to be a sequence of i.i.d. random variables such. We have ℋ(s,a) as a random variable representing the number of terms in the product, independent of $T_{t}$, and assume ℋ(s,a)∈N. Therefore, the sum of the logarithms $\sum_{t=0}^{{ℋ}^{*}\left(s,a\right)}\ln T_{t}$ approximately follows a normal distribution under the following conditions:

1. Finite Expectation and Variance of $\ln T_{t}$: The expectation $E[\ln T_{t}]$ depends on the distribution of $T_{t}$, with a higher probability density near 1 leading to a less negative mean. The variance $\mathrm{Var}(\ln T_{t})$ is finite and captures the spread of $\ln T_{t}$:
  we know that for sure that $T_{t}\in \left(a,1\right]$, with 0≪a≤1 since this transition is $\max_{s,a}T\left(s,a,s^{\prime }\right)$. Such behavior is characteristic of deterministic or near-deterministic environments, which are common in many RL tasks. Based on this observation, we posit that RL environments of interest adhere to this description, thereby satisfying the condition of finite expectation and variance for $\ln T_{t}$.
2. Sufficiently Large $E[{ℋ}^{*}\left(s,a\right)]$: When *Y* is large enough (e.g., $E[{ℋ}^{*}\left(s,a\right)]\gg 1$), the sum $\sum_{t=0}^{ℋ(s,a)}\ln T_{t}$ satisfies the central limit theorem, approximating a normal distribution due to the aggregation of independent terms.

**Edge Case**: if ${ℋ}^{*}\left(s,a\right)$ is small, the normality of $\sum_{t=1}^{{ℋ}^{*}\left(s,a\right)}\ln T_{t}$ may break down. This happens when the state-action pair in question is often close to the goal, leading to fewer steps to the goal. In such cases, breaking the normality is not problematic, because the values of value function around these states are overall high and so is the expectation from this set of values, which is easy for the agent to converge during learning according to optimistic initialization concept. Consequently, this edge case does not interfere with the objective of value function initialization. □

### Appendix A.4. Environment Description

Mountain car: to learn it in a tabular fashion we discretized the state position and velocity interval into 30 × 30. We used a binary reward where zero is assigned to non rewarding states and 1 to achieving the goal in order to fit into the logarithm properties used by LogQInit. the learning rate alpha is α=0.05 and the exploration method we used is ϵ-greedy where ϵ=0.5 during learning tasks without transfer, and ϵ=0.1 for tasks with receiving knowledge transfer.
